# Antibiotic De-Escalation in Critically Ill Patients with Negative Clinical Cultures

**DOI:** 10.3390/pharmacy11030104

**Published:** 2023-06-16

**Authors:** Spencer Roper, Mary Joyce B. Wingler, David A. Cretella

**Affiliations:** 1 Department of Pharmacy, University of Tennessee Medical Center, Knoxville, TN 37920, USA; 2 Department of Antimicrobial Stewardship, University of Mississippi Medical Center, Jackson, MS 39216, USA

**Keywords:** antibiotic de-escalation, intensive care unit, antimicrobial stewardship

## Abstract

(1) Background: Antibiotics are received by a majority of adult intensive care unit (ICU) patients. Guidelines recommend antibiotic de-escalation (ADE) when culture results are available; however, there is less guidance for patients with negative cultures. The purpose of this study was in investigate ADE rates in an ICU population with negative clinical cultures. (2) Methods: This single-center, retrospective, cohort study evaluated ICU patients who received broad-spectrum antibiotics. The definition of de-escalation was antibiotic discontinuation or narrowing of the spectrum within 72 h of initiation. The outcomes evaluated included the rate of antibiotic de-escalation, mortality, rates of antimicrobial escalation, AKI incidence, new hospital acquired infections, and lengths of stay. (3) Results: Of the 173 patients included, 38 (22%) underwent pivotal ADE within 72 h, and 82 (47%) had companion antibiotics de-escalated. Notable differences in patient outcomes included shorter durations of therapy (*p* = 0.003), length of stay (*p* < 0.001), and incidence of AKI (*p* = 0.031) in those that underwent pivotal ADE; no difference in mortality was found. (4) Conclusions: The results from this study show the feasibility of ADE in patients with negative clinical cultures without a negative impact on the outcomes. However, further investigation is needed to determine its effect on the development of resistance and adverse effects.

## 1. Introduction

Most patients in adult intensive care units (ICUs) receive antibiotics for suspected or confirmed infection. A 2009 study found that ICUs across numerous countries reported that 51% of their patients had confirmed infection, and 71% were receiving antibiotics [1]. While early antimicrobial therapy is crucial for critically ill patients with suspected infection [2], prolonged antibiotic exposure increases the risk for the development of antimicrobial resistance [3]. A 2019 study of patients receiving broad-spectrum beta-lactams, including cefepime, piperacillin/tazobactam, and meropenem, found that each additional day of therapy increased the risk of resistance development by 8% [3]. In order to prevent resistance development, the Infectious Diseases Society of America (IDSA) guidelines recommend antimicrobial de-escalation (ADE) of empirical therapy on the basis of culture results, in order to limit broad-spectrum antimicrobial exposure [4]. Other benefits of limiting the unnecessary or inappropriate use of antimicrobials include the decrease in incidence of adverse effects, such as acute kidney injury and secondary infections, which can prolong hospital stay.

In 2020, the European Society of Intensive Care Medicine (ESICM) and the European Society of Clinical Microbiology and Infectious Diseases (ESCMID) released a position statement on ADE in critically ill patients [5]. This task force suggested that empirical regimens can safely be de-escalated without negatively impacting patients’ clinical outcomes based on the available data. The task force’s definition of ADE in critically ill patients included either (1) narrowing the spectrum of activity or lowering the ecological impact or (2) stopping double coverage or coverage for a pathogen not isolated in cultures, such as methicillin-resistant *Staphylococcus aureus* (MRSA). In addition to creating a standardized definition, an algorithm was developed to assist with ADE, which separated antibiotics into two categories: pivotal and companion antibiotics. Pivotal antibiotics were defined as the central agent to the regimen, which is typically a beta-lactam antibiotic with Gram-negative coverage. Companion antibiotics could include agents used to broaden the spectrum of coverage of the pivotal antibiotic, such as anti-MRSA agents. For patients with positive cultures, it is suggested to perform ADE within 24 h of definitive culture results, and previous studies have shown that ADE appears to be more common in patients with microbiological documented infections [6]. However, in patients with culture-negative infections, the task force recommends evaluating for alternative non-infectious diagnosis and stopping all antibiotics, or performing ADE if discontinuing antibiotics is not possible.

Though ESICM/ESCMID provides some guidance on patients with culture-negative infections, de-escalating antimicrobial therapy in these patients remains a challenge. Clinicians often feel hesitant de-escalating broad-spectrum antibiotics without microbiology to guide them, even with evidence of clinical improvement. As an example, one large study conducted by Desphande and colleagues limited their evaluation of ADE in non-critically ill patients with pneumonia specifically to patients with negative cultures. This study highlighted provider hesitancy upon de-escalation in culture-negative patients, with an overall de-escalation rate of only 13% across 164 hospitals. Despite low de-escalation rates, an improvement in patient outcomes was found in those who successfully underwent de-escalation [7]. IDSA guidelines offer no formal recommendations on the de-escalation process when cultures are negative. These guidelines recognize the urgent need for more studies assessing the effect of de-escalation on outcomes in culture-negative patients [4]. The purpose of this study was to evaluate an academic medical center’s rates of de-escalation in the ICU for patients with negative cultures, and compare the differences in the outcomes between patients who had their antibiotics de-escalated within 72 h and those that did not.

## 2. Materials and Methods

### 2.1. Study Design and Study Setting

This single-center, retrospective, cohort study evaluated patients admitted to the 100-bed ICU tower of an academic medical center from September 2018 until December 2019. Eligible patients were ≥18 years old, received a broad-spectrum antibiotic regimen (defined below) within 24 h of ICU admission, had a total duration of antibiotic therapy of 5 days or greater, and had negative clinical cultures. Patients were excluded if they received antibiotics for peri-operative prophylaxis, esophageal/bowel perforations, facial trauma or penetrating brain injury, febrile neutropenia, or acute exacerbations of cystic fibrosis. If patients received more than one antibiotic course upon the same admission, only the first antibiotic course of ≥5 days was included. This study was approved by the medical center’s Institutional Review Board (protocol number #2020V0252).

Patients with negative clinical cultures were separated into two study groups: those that underwent pivotal drug de-escalation or those that did not undergo pivotal drug de-escalation. Once the patients were stratified into their respective study groups, relevant data were collected on the password-protected database, REDCap [8]. The following data points were recorded: patient demographics, comorbidities, hospital and ICU length of stay, ICU the patient was admitted to, APACHE II score on day 1 of antibiotics, serum creatinine changes, site of presumed infection, pivotal antibiotics started in ICU, companion antibiotics started in ICU, alterations in antibiotic therapy, days of antibiotic therapy, culture results, times between antibiotic initiation and culture obtainment, MRSA PCR results, ID consult, hospital-acquired infections (HAI) within 30 days of antibiotic completion, HAI pathogens, *Clostridiodes difficile* infections, ICU all-cause mortality, and all-cause mortality within 30 days of ICU discharge.

### 2.2. Definitions

Negative clinical cultures were defined as those finalizing with no growth or no evidence of a single offending pathogen, including normal flora (e.g., “normal respiratory flora”). These patients with negative clinical cultures were also evaluated for culture appropriateness based on the site of the presumed infection. At a minimum, the patients had to have had the following cultures drawn depending on their site of presumed infection: pneumonia = blood and respiratory; urinary tract infection (UTI) = blood and urine; abdominal infection = blood and wound; skin and soft tissue infection (SSTI) = blood; sepsis of unknown source = blood. Blood and urine cultures were considered appropriate if obtained within 24 h of antibiotic initiation. Respiratory cultures had to be obtained within 48 h of antibiotic initiation. The site of the presumed infection was assessed as defined by The International Sepsis Forum Consensus Conference [9].

For the purposes of this study, a broad-spectrum regimen was one consisting of at least one beta-lactam antimicrobial active against *Pseudomonas aeruginosa* and either an antimicrobial with MRSA coverage for a suspected MRSA infection or double *P. aeruginosa* coverage. Pivotal antibiotics were the anti-pseudomonal beta-lactams, including piperacillin/tazobactam, cefepime, ceftazidime, and meropenem. The companion antibiotics were the agents added for MRSA coverage (vancomycin, linezolid) or double *P. aeruginosa* coverage (aminoglycosides, fluoroquinolones).

Patients were considered to have undergone de-escalation if antibiotics were narrowed in spectrum within 72 h of therapy initiation. During the study period, there was no institutional protocol for de-escalation. Adapted from the Duke Antimicrobial Stewardship Outreach Network (DASON) antibiotic rankings, pivotal antibiotics were stratified into different groups based on the spectrum of activity and ability to cover *P. aeruginosa* [10]. Pivotal antibiotic de-escalation was defined as moving down in the group (e.g., going from meropenem to cefepime). These groupings were as follows:

- Group 1: amoxicillin, ampicillin, amoxicillin/clavulanate, ampicillin/sulbactam, ceftriaxone, cefpodoxime, cefuroxime, cefdinir, cephalexin;
- Group 2: piperacillin/tazobactam, cefepime, ceftazidime, aztreonam, levofloxacin, ciprofloxacin;
- Group 3: meropenem.

Companion antibiotic de-escalation occurred if the antibiotic was discontinued completely. As part of data collection, the incidence of antibiotic escalation was evaluated as well, and was defined as either switching from one pivotal antibiotic to another in a higher group or an addition to antibiotic therapy without de-escalation (did not include additions for atypical, anaerobic, fungi, or virus coverage).

### 2.3. Statistical Analysis

Categorical data were assessed using chi-square or Fisher’s exact test, and continuous data were assessed using Student’s *t*-test or the Mann–Whitney U test, as appropriate. No imputation was made for any missing data. Statistical analysis was performed via SPSS 27 (IBM, Armonk, NY, USA, 2019) and statistical significance was defined as *p* < 0.05.

## 3. Results

Nine hundred and sixty-one patients were screened for enrollment, yielding 173 patients who could be included. The most common reasons for exclusion were either positive cultures or not receiving at least 5 days of antimicrobial therapy. Of the 173 patients included in this study, 38 patients (22.0%) underwent pivotal antibiotic de-escalated within the first 72 h following antimicrobial initiation. Companion antibiotics were discontinued by 72 h in 82 patients (47.4%), and 81/82 (98%) of the companion antibiotics were anti-MRSA agents. Patients who underwent pivotal antibiotic de-escalation were more likely to no longer require vasopressors (*p* = 0.010). A full list of demographics can be seen in Table 1.

The most common indication for antibiotics was pneumonia (50.9%) (Table 2). Piperacillin/tazobactam was the most common empirically selected pivotal antibiotic (71.7%). One hundred and six patients (61.3%) had appropriate cultures drawn for the diagnosed infection, and most patients had cultures obtained on the day antibiotics were initiated (Table 3). Respiratory cultures were only obtained in 58% of patients with pneumonia, and this was not statistically different between those who did and did not undergo de-escalation. MRSA PCR utilization for the purpose of vancomycin de-escalation was higher in the de-escalation population (*p* = 0.016), and positivity for the MRSA PCR was low in both groups. Only 15% of patients had their antibiotics managed by an Infectious Disease team.

Patients who underwent pivotal ADE had shorter hospital length of stay (*p* < 0.001), ICU length of stay (*p* < 0.001), and durations of antibiotic therapy (*p* = 0.003), and a lower incidence of AKI (*p* = 0.031). No statistical difference was found for the development of antibiotic resistance. The remaining secondary outcomes can be found in Table 4.

## 4. Discussion

In this single-center, retrospective study, the rate of pivotal ADE at 72 h was low (22.0%). The baseline characteristics were balanced between the groups, with most patients presenting with comorbidities, similar APACHE II scores, and community-acquired infections. The rate of companion antibiotic de-escalation was higher (47.4%), potentially due to the availability of rapid diagnostic testing for MRSA. Patients who underwent de-escalation were more likely to have vasopressors discontinued by day 3, which may have played a role in the decision to change antibiotic therapy. In addition, ADE did not appear to be correlated with any negative outcomes. Patients undergoing de-escalation did not have a higher mortality, but had a significantly shorter length of stay and duration of antibiotic therapy.

The low rate of de-escalation found in our study is similar to what has been described in previous literature. One of the largest studies evaluating ADE in critically ill patients was the DetermInants of Antimicrobial use aNd de-escalAtion (DIANA) trial [6]. This multicenter, prospective, observational study included 1495 patients, and only 240 (16%) has ADE performed in the first 3 days of antimicrobial therapy. Of these 240 patients, most patients (52%) had one or more of the antibiotics used in combination therapy discontinued (i.e., companion antibiotic ADE). Eighty-four patients (35%) had the antibiotic agent replaced by another drug (i.e., pivotal antibiotic ADE). The clinical outcomes were not negatively impacted by ADE, and patients who underwent ADE had higher rates of clinical cure, shorter LOS, and similar rates of mortality. The DIANA study included patients with microbiologically confirmed infection and those with negative cultures, but ADE seemed more likely to occur in patients with positive cultures. A smaller study conducted by Routsi and colleagues specifically evaluated the rate of ADE in intensive care units with high rates of antimicrobial resistance, and found similar results. A total of 262 critically ill patients with a documented infection were included, but 97 (37%) had drug-resistant pathogens. Of the remaining 165 (63%) patients eligible for ADE, only 60 (23%) had antibiotics de-escalated. [11]. Desphande and colleagues explored de-escalation in patients diagnosed with pneumonia who exclusively had negative cultures [7]. This multicenter study evaluated de-escalation rates across 164 US hospitals and included 14,170 patients. Overall, the de-escalation rate was 13%, but this ranged from 2 to 35% across the different hospitals. There was no difference in mortality between patient who underwent de-escalation by day 4 and those that continued broad-spectrum antibiotics (*p* = 0.095). De-escalation was also found to be associated with reductions in all negative outcomes (ICU admission rates, LOS, hospitalization costs, etc.), except *C. difficile* infections. Most hospitals included in this study had de-escalation rates lower than ours, despite only including non-critically ill patients, but the outcomes were similar.

While several previous publications show similar de-escalation rates to ours, other smaller studies do exhibit higher rates of de-escalation. Liu and colleagues found that 63% of patients had antibiotics de-escalated at 72 h [12]. The higher rate of de-escalation in their study may have been due to the population included, which were non-critically ill patients and those with positive and negative cultures. However, at least two studies in ICU patients have also demonstrated high rates of de-escalation [13,14]. Trupka and colleagues performed a prospective cross-over trial targeted at ADE in 283 medical ICU patients with suspected pneumonia [13]. Antibiotics were either managed by a critical care team (routine antibiotic management) or reviewed by the study team and recommendations were made (enhanced antibiotic de-escalation). High rates of ADE occurred in both groups at 67% and 66%, respectively. Another study retrospectively assessed 113 ICU patients with hospital-acquired, ventilator-associated, or healthcare-associated pneumonia, and found an ADE rate of 62% [14]. Across these two studies, the clinical outcomes were similar or better in patients who had ADE performed. In addition, Knaack and colleagues found that in-hospital mortality, LOS, and hospitalization costs were significantly lower in patients who had antibiotics de-escalated [14]. One similarity amongst these studies with high ADE rates is the source of infection, with the studies either only evaluating ADE in patients with pneumonia or the majority of patients in the study had pneumonia. Likewise, most patients in our study had pneumonia and may be high-yield targets for de-escalation at our institution. 

Given the variable and sometimes low rate of ADE in critically ill patients, it is important to identify the predictors of ADE. One important factor in promoting ADE appears to be microbiologic culture results [15,16,17]. An analysis of 113 meropenem courses in 67 surgical ICU patients found an overall ADE rate of 42%. However, 79% of those who underwent ADE had conclusive microbiologic results, compared to 44% of those that did not undergo ADE (*p* < 0.01) [15]. Similarly, a study of 115 patients with ventilator-associated pneumonia found an overall ADE rate of 31% [16]. Ten patients had no microorganism identified upon culture, and none had ADE performed. In addition, de-escalation was found to occur less frequently in the presence of nonfermenting Gram-negative rods (3% vs. 49%) and in the presence of late-onset pneumonia (13% vs. 41%). Heenen and colleagues performed a review of 169 patients with severe sepsis in an academic medical-surgical ICU [17]. They evaluated not only the rate and extent of ADE, but also the rationale supporting or preventing ADE. De-escalation occurred in 43% of patients. When evaluating the patients that did not undergo ADE, 46% could not have been narrowed due to microbiologic sensitivities. Among the remaining patients, 32% did not undergo ADE due to lack of culture data and 13% did not undergo ADE because microbiologic data were considered inconclusive or unreliable. Only 5% of the patients had no identifiable reason for not undergoing ADE. Based on this, they concluded that in critically ill ICU patients, high rates of ADE may be unachievable. There is also evidence that that the way in which microbiologic results are reported can affect the rate of ADE [18]. In a study of 210 hospitalized patients receiving an anti-staphylococcal and anti-pseudomonal antibiotics, the implementation of a microbiology comment “nudge”, noting the absence of *S. aureus* and *P. aeruginosa* in respiratory cultures growing “commensal respiratory flora”, led to a significant increase in ADE [18]. Because microbiological results are likely a large driver of successful ADE, culture obtainment may have impacted the rate of ADE in this study. For example, patients with pneumonia had a low rate of respiratory culture obtainment in both groups. Both the community-acquired pneumonia and hospital-acquired/ventilator-associated pneumonia guidelines recommend obtaining respiratory cultures for severe pneumonia [19,20]. The de-escalation of broad-spectrum antibiotics is challenging without respiratory cultures; therefore, this may indicate that additional education is needed regarding culturing practices, particularly for lower respiratory tract infections. MRSA PCR was collected and while it was most likely used for isolation purposes earlier in the study period, providers may have begun using it for diagnostic reasons as more evidence of its diagnostic utility has emerged. In the absence of appropriate cultures, physicians may have been more comfortable with de-escalation in patients with negative MRSA PCRs.

When evaluating the potential negative effects associated with not performing ADE, our study found a statistically significant difference in the development of AKI, with patients undergoing de-escalation demonstrating a decreased rate of AKI. This, in part, could be attributed to the withdrawal of singular nephrotoxic agents or the de-escalation of nephrotoxic combination therapy. For instance, the high rate of piperacillin–tazobactam and vancomycin used in this cohort may contribute to this correlation, given that the combination is associated with AKI [21]. There was a trend toward a high incidence of the development of a new *C. difficile* infection in patients that continued their initial regimen, but this was not statistically significant. Another outcome evaluated was the development of resistance because there is evidence suggesting that the use of broad-spectrum antibiotics is associated with this outcome, as described above. [3] However, there are limited data on how ADE impacts antimicrobial resistance. One retrospective study found no decrease in the emergence of antibiotic resistance following de-escalation of anti-pseudomonal beta-lactam antibiotics [22]. Other studies have described similar results, but most had relatively small populations and may not have been able to detect a difference [6,23]. We found no difference in the development of resistance between patients who underwent ADE and those who did not. The ESICM/ESCMID position statement gave no recommendation due to the inconclusiveness of current data, and additional studies are needed to evaluate this question.

One concern associated with de-escalation is that some evidence suggests that de-escalation may be associated with an increased duration of therapy [24]. Leone and colleagues evaluated ADE in patients with severe sepsis in nine ICUs in France. Antibiotic days in the de-escalation group was 9 compared with 7.5 in the de-escalation group (*p* = 0.03). In contrast, data gathered in our study show that patients who underwent de-escalation had a statistically significant decrease in their total duration of antimicrobial therapy. One reason for this could be explained by more patients having been diagnosed as having sepsis of an unknown source in the non-ADE arm. Without a defined source, it may have been more challenging for providers to perform ADE.

Finally, a question remains on the best time to perform ADE in critically ill patients. We evaluated ADE within 72 h after antibiotic initiation, since that is generally when culture results are available. In patients with positive cultures, de-escalation could be considered even sooner. If rapid diagnostics are available, these tests can not only facilitate organism identification, but also the presence of resistance for some tests, such as PCR-based blood culture identification panels. The MRSA PCR is another example of a test that has been implemented at many institutions as a tool for de-escalation. For example, Mergenhagen and colleagues previously found that the negative predictive value (NPV) of MRSA PCR screening used to rule out invasive MRSA infection was 96.5%, describing their utility with de-escalation and empiric avoidance [25]. Outside of MRSA PCR, other multiplex PCRs for lower respiratory tract infections (LRTI) show promising data for detecting bacteria and resistance genes from brocheo-alveolar lavage fluid. Klein and colleagues showed that their multiplex PCR had an overall positive predictive value (PPA) and negative predictive value (NPV) of 93.4% and 98.3%, respectively [26]. Another retrospective study using the same PCR test found that the utilization of results from the PCR could have potentially changed the management of 87.6% of patients [27].

This study did have important limitations. This is a small, single-center study, limiting generalizability to other centers. In addition, the retrospective nature of the study introduces potential selection bias. As previously mentioned, patients with earlier improvement (vasopressors discontinued by day 3) were more likely to undergo ADE, which may be why clinical outcomes were similar between the groups. Multiple infection types were included, but pneumonia was the most common; therefore, one should exercise caution when applying the results of this study to less represented disease states. No risk factors for MRSA or *P. aeruginosa* were assessed to ensure that patients were indicated for broad-spectrum antibiotics empirically. During the study period, antimicrobial stewardship and ICU pharmacist presence increased, which may have affected de-escalation rates in the latter years.

## 5. Conclusions

ADE of empiric broad-spectrum antimicrobials is recommended by experts, clinical practice guidelines, and professional organizations. However, ADE remains difficult and infrequent in critically ill patients, particularly those without positive clinical cultures. This study adds to the current literature regarding ADE in this population. Our results showed that ADE was rare, but was associated with shorter length of stay and duration of therapy, and lower incidences of AKI, without increased mortality. One area for improvement identified was the obtainment of cultures, and providing education should be prioritized going forward in order to assist with de-escalation. Further study is warranted to corroborate these findings, explore the impact on resistance development, and determine the applicability of these findings to infectious states other than pneumonia. Health systems should continue to advocate for ADE in patients, including those who are critically ill, to prevent adverse drug events and the development of antimicrobial resistance.

## Figures and Tables

**Table 1:** Baseline characteristics.

Variable	Pivotal Antibiotic De-Escalation (n = 38)	No Pivotal Antibiotic De-Escalation (n = 135)	*p*-Value
Age, mean (SD)	59.5 (±17.8)	56.4 (±16.6)	0.699
Race, n (%)			
Black	20 (52.6)	75 (55.6)	0.854
White	18 (47.4)	53(39.3)	0.456
Other	0	7 (5.2)	1.000
Comorbidities, n (%)			
Hypertension	22 (57.9)	74 (54.8)	0.736
Heart Failure	7 (18.4)	29 (21.5)	0.681
Diabetes Mellitus	11 (28.9)	40 (29.6)	0.935
COPD	4 (10.5)	17 (12.6)	1.000
History of CVA	3 (7.9)	15 (11.1)	0.766
Moderate–Severe Liver Disease	1 (2.6)	4 (3.0)	1.000
CKD Stage III-V	3 (7.9)	11 (8.1)	1.000
End-stage Renal Disease	0	12 (8.9)	0.071
No Listed Comorbidities	10 (26.3)	38 (28.1)	0.824
Unit Responsible for Patient Care, n (%)			
SICU	1 (2.6)	14 (10.4)	0.196
MICU	31 (81.6)	77 (57.0)	0.006
CCU	4 (10.5)	29 (21.5)	0.129
NSICU	2 (5.3)	15 (11.1)	0.369
Mechanically Ventilated, n (%)	17 (44.7)	75 (55.6)	0.238
Vasopressors—Day 1, n (%)	12 (31.6)	54 (40.0)	0.345
Vasopressors—Day 3, n (%)	5 (13.2)	47 (34.8)	0.010
Community-Acquired Infection, n (%)	32 (84.2)	102 (75.6)	0.259
ID Consult, n (%)	6 (15.8)	20 (14.8)	0.882
APACHE II Score, mean (SD)	14.37 (±5.73)	16.08 (±6.86)	0.227

SD: standard deviation; COPD: chronic obstructive pulmonary disease; CVA: cardiovascular accident; CKD: chronic kidney disease; SICU: surgical intensive care unit; MICU: medical intensive care unit; CCU: cardiovascular care unit; NSICU: neurosurgical intensive care unit; ID: Infectious Disease; APACHE: Acute Physiology and Chronic Health Evaluation.

**Table 2:** Antibiotic and infection characteristics.

Variable	All (N = 173)	Pivotal Antibiotic De-Escalation (n = 38)	No Pivotal Antibiotic De-Escalation (n = 135)	*p*-Value
Site of Presumed Infection, n (%)				
Pneumonia	88 (50.9)	23 (60.5)	65 (48.1)	0.178
Abdominal Infection	14 (8.1)	1 (2.6)	13 (9.6)	0.309
Skin and Soft Tissue Infection	11 (6.4)	2 (5.3)	9 (6.7)	1.000
Urinary Tract Infection	9 (5.2)	6 (15.8)	3 (2.2)	0.004
Sepsis of Unknown Source	45 (26.0)	3 (7.9)	42 (31.1)	0.004
Other	6 (3.5)	3 (7.9)	3 (2.2)	0.121
Pivotal Antibiotic Used, n (%)				
Piperacillin/Tazobactam	124 (71.7)	29 (76.3)	95 (70.4)	0.472
Cefepime	43 (24.9)	5 (13.2)	38 (28.1)	0.059
Meropenem	6 (3.5)	4 (10.5)	2 (1.5)	0.022

**Table 3:** Diagnostics.

Variable	All (N = 173)	Pivotal Antibiotic De-Escalation (n = 38)	No Pivotal Antibiotic De-Escalation (n = 135)	*p*-Value
Blood culture obtained within 24 h, n (%)	147 (85.0)	36 (94.7)	111 (82.2)	0.057
Appropriate culture obtained, n (%)	106 (61.3)	23 (60.5)	83 (61.5)	0.915
MRSA PCR performed, n (%)	98 (56.6)	28 (73.7)	70 (51.9)	0.016
Positive MRSA PCR results, n (%)	9 (9.2)	2 (7.1)	7 (10.0)	1.000

MRSA: methicillin-resistant *Staphylococcus aureus*; PCR: polymerase chain reaction.

**Table 4:** Secondary outcomes.

Variable	All (N = 173)	Pivotal Antibiotic De-Escalation (n = 38)	No Pivotal Antibiotic De-Escalation (n = 135)	*p*-Value
New HAI, non-*C. difficile*, n (%)	30 (17.3)	4 (10.5)	26 (19.3)	0.209
Escalation of therapy, n (%)	21 (12.1)	6 (15.8)	15 (11.1)	0.412
*C. difficile* infection, n (%)	6 (3.5)	1 (2.6)	5 (3.7)	1.000
Incidence of AKI, n (%)	41 (23.7)	4 (10.5)	37 (27.4)	0.031
All-cause, ICU mortality, n (%)	24 (13.9)	3 (7.9)	21 (15.6)	0.227
All-cause, inpatient mortality, n (%)	32 (18.5)	6 (15.8)	26 (19.3)	0.627
Length of stay in days, median (IQR)	15 (9.0–26.0)	9 (7.3–15.0)	17 (10.0–29.5)	<0.001
Length of ICU stay in days, median (IQR)	7 (4.0–14.0)	5 (3.0–7.0)	9 (5.0–16.0)	<0.001
Days of pivotal antibiotic therapy, median (IQR)	5 (4.0–7.0)	3 (2.0–4.0)	6 (5.0–8.0)	<0.001
Days of MRSA companion antibiotic therapy, median (IQR)	4 (2.0–7.0)	3 (1.3–3.0)	5 (2.0–7.0)	<0.001
Total days of antibiotic therapy, median (IQR)	7 (6.0–10.0)	6 (5–7.8)	8 (6.0–10.0)	0.003

HAI: hospital-acquired infection; AKI: acute kidney injury; ICU: intensive care unit; IQR: interquartile range; MRSA: methicillin-resistant *Staphylococcus aureus.*

## Data Availability

Not applicable.
